# Metabolic disruption identified in the Huntington’s disease transgenic sheep model

**DOI:** 10.1038/srep20681

**Published:** 2016-02-11

**Authors:** Renee. R. Handley, Suzanne J. Reid, Stefano Patassini, Skye R. Rudiger, Vladimir Obolonkin, Clive. J. McLaughlan, Jessie C. Jacobsen, James F. Gusella, Marcy E. MacDonald, Henry J. Waldvogel, C. Simon Bawden, Richard L. M. Faull, Russell G. Snell

**Affiliations:** 1 Centre for Brain Research, University of Auckland, Auckland, 1010, New Zealand; 2 Molecular Biology and Reproductive Technology Laboratories, South Australian Research and Development, Adelaide, SA 5350, Australia; 3 Research & Development, Livestock Improvement Corporation, Hamilton, 3240, New Zealand; 4 Center for Human Genetic Research, Massachusetts General Hospital, Harvard Medical School, Boston MA 02114, United States of America

## Abstract

Huntington’s disease (HD) is a dominantly inherited, progressive neurodegenerative disorder caused by a CAG repeat expansion within exon 1 of *HTT*, encoding huntingtin. There are no therapies that can delay the progression of this devastating disease. One feature of HD that may play a critical role in its pathogenesis is metabolic disruption. Consequently, we undertook a comparative study of metabolites in our transgenic sheep model of HD (OVT73). This model does not display overt symptoms of HD but has circadian rhythm alterations and molecular changes characteristic of the early phase disease. Quantitative metabolite profiles were generated from the motor cortex, hippocampus, cerebellum and liver tissue of 5 year old transgenic sheep and matched controls by gas chromatography-mass spectrometry. Differentially abundant metabolites were evident in the cerebellum and liver. There was striking tissue-specificity, with predominantly amino acids affected in the transgenic cerebellum and fatty acids in the transgenic liver, which together may indicate a hyper-metabolic state. Furthermore, there were more strong pair-wise correlations of metabolite abundance in transgenic than in wild-type cerebellum and liver, suggesting altered metabolic constraints. Together these differences indicate a metabolic disruption in the sheep model of HD and could provide insight into the presymptomatic human disease.

Huntington’s disease (HD) is a dominantly inherited neurodegenerative disorder characterised by variable onset of motor dysfunction, cognitive decline and psychiatric disturbance. HD is caused by the expansion of a CAG repeat sequence within exon 1 of *HTT*, resulting in an expanded polyglutamine tract (>38 units) in the ubiquitously expressed huntingtin protein^1^. There is currently no treatment that prevents onset or delays disease progression. The development of effective disease-modifying therapies requires a better understanding of the early or primary molecular pathogenic process. The identification of biomarkers with which to track disease progression is also important for therapeutic testing.

In recent years, hypothesis-free profiling methods have provided a plethora of new observations characterising the molecular changes that occur in HD. Microarray analysis of post-mortem HD brain revealed region-specific changes in the expression of genes involved in neurotransmitter signalling, calcium homeostasis, axonal transport, gliosis and neuro-inflammation^2^. RNA-Seq has identified altered expression of micro-RNAs in the frontal cortex and striatum^3^. Metabolite analysis via Nuclear Magnetic Resonance spectroscopy and gas-chromatography mass spectrometry have revealed altered levels of amino acids in blood and what was considered a hyper-metabolic phenotype^4^,^5^.

Metabolic dysfunction in HD is observed as both gross phenotypic and molecular effects. Individuals with HD have a lower body mass index than unaffected individuals^6^, which declines prior to the onset of other symptoms^7^,^8^, despite increased caloric intake^9^,^10^. A cellular energy deficit is thought to underlie this phenotype. Indeed, whole indirect calorimetry reveals that HD patients are in a state of negative energy balance even early in the disease^11^ and declining levels of branched chain amino acids in plasma from HD patients have been specifically shown to inversely correlate with weight loss^10^.

The utilisation rate of glucose in the HD striatum is reduced compared to unaffected individuals^12^, cholesterol and fatty acid synthesis is impaired^13^, and levels of adenosine triphosphate (ATP) relative to adenosine diphosphate (ADP) are altered in both brain and peripheral tissues^14^. The latter cellular energy-charge inversely correlates with the length of the CAG repeat in cultured lymphoblastoid cells^14^; the CAG repeat in turn has a well-known inverse correlation with age at onset. This functional link of the HD mutation to fundamental metabolic regulation, together with the early and progressive presentation of metabolic phenotypes in patients, makes addressing the metabolic defect in HD an attractive therapeutic approach.

Although the pre-manifest period of HD is likely to be the best therapeutic window, our knowledge of molecular changes in this phase is limited. In order to study the natural history of HD as a late onset disorder, including progression of pathogenesis through the pre-manifest period, our laboratory developed a transgenic sheep model^15^. The OVT73 sheep line expresses a full length human huntingtin cDNA with a pathogenic polyglutamine-coding repeat of 73 units, inserted within a single locus of the sheep genome^16^,^17^. Molecular changes that are characteristic of the HD brain are detected in the OVT73 sheep, including huntingtin-positive neuropil and intra-nuclear neuronal aggregates in cortical tissue and reduced staining of gamma-aminobutyric acid receptor alpha 1 in the striatum^17^. Despite these pathologies the OVT73 sheep show no obvious neuronal loss and are outwardly indistinguishable from wild-type sheep, except for a circadian rhythm abnormality^18^, indicating that they may be a good model of pre-manifest HD.

Metabolite profiling has been undertaken in rodent models of HD, revealing altered levels of cholesterol synthesis precursors in R6/1 mouse brain tissue from 6 weeks of age^19^ and reduced N-Acetylaspartate (NAA), aspartate and alanine in the striatum of late-symptomatic R6/2 mice^20^. Reduced levels of NAA, have also been reported following H-NMR analysis of serum from presymptomatic rats carrying a transgene with a CAG repeat expansion of 51 units, supporting an early impairment of mitochondrial function in HD^21^. Findings from these models are interesting, but must be considered in the context of the truncated mutant huntingtin construct expressed in both models as well as the severity of the phenotype exhibited by the R6/2 miced. Metabolite profiling of animal models carrying full length mutant huntingtin may provide more biologically relevant insight into the metabolic defect.

To investigate metabolic changes in an animal model expressing full length mutant huntingtin and before overt symptoms or neuropathology of HD we performed a comparative analysis of metabolite profiles of brain tissue (cerebellum, motor cortex and hippocampus) from OVT73 and wild-type sheep, using gas chromatography-mass spectrometry (GC-MS). The brain regions studied were readily available in our tissue bank and selected to broadly represent cortical and subcortical structures with cognitive and motor functions. Although the striatum reveals the most severe pathology in HD, unfortunately there was insufficient striatal tissue available for analysis. To investigate whether a metabolic defect in HD may be systemic rather than brain-specific we also performed GC-MS analysis of liver tissue from the sheep.

Differences in metabolite abundance were detected that indicate the existence of a metabolic defect in the OVT73 sheep and could provide insight into the pre-symptomatic human disease[Table t1].

## Results

### Composition of the Sheep GC-MS Metabolite Profiles

Metabolite profiles were generated by GC-MS for each of 4 tissue types dissected from 6 wild-type and 6 OVT73 sheep (sample details are provided in Table 1). Processing the data through the automated pipeline *Metab* identified and quantified 51 metabolites in the cerebellum, 37 in the hippocampus, and 48 in each of the motor cortex and liver in both wild-type and OVT73. The identified compounds included fatty acids and amino acids/amino acid derivatives in approximately equal proportions, with a smaller group of other organic acids also detected (Table 2). Differences in the abundance of identified metabolites were observed in the OVT73 cerebellum and liver, but not in hippocampus or motor cortex, compared to wild-type tissues (reported below).

### Differential Abundance of Single Metabolites in the OVT73 Cerebellum

**Figure 1:**
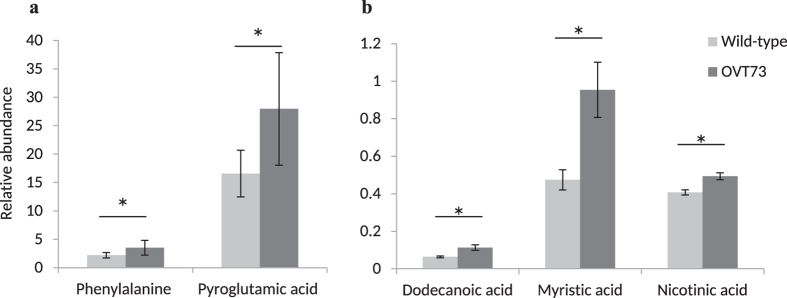
Differential Abundance of Single Metabolites in OVT73 Cerebellum and Liver GC-MS metabolite analysis of cerebellum and liver in transgenic (n = 6) and wild-type (n = 6) sheep. Metabolite abundance is expressed relative to the abundance of 2,3,3,3-d_4_-alanine internal standard and back-transformed. (**a**) Phenylalanine and pyroglutamic acid abundance in cerebellum (**b**) Dodecanoic acid, myristic acid and nicotinic acid abundance in liver. False Discovery Rate adjusted *P* < 0.05*, Standard Least Squares. Error bars are the standard error of the mean back-transformed relative abundance.

The average relative abundance of 11 metabolites differed with nominal significance in the OVT73 cerebellum compared with wild-type (Table 3). All 11 metabolites were more abundant in the OVT73 cerebellum, and 9 of the 11 metabolites were amino acids/amino acid derivatives. Differences in the relative abundance of 2 metabolites; pyroglutamic acid (69%) and phenylalanine (61%), remained significant after FDR adjustment for multiple testing (Fig. 1).

As the samples used in this study were an unbalanced cohort of rams (males) and ewes (females) we investigated effects of sex on the 11 metabolites that were differentially abundant in the OVT73 cerebellum. Ten of the 11 metabolites remained differentially abundant (*P* < 0.05) in a statistical model where the variable of transgene status was nested within the variable of sex. Differences in the abundance of 8 metabolites were more significant in the nested model than for the transgene effect alone, indicating an interaction between sex and transgene status (Table 4). Examining the effect of transgene status for each sex respectively, all 11 metabolites were differentially abundant between OVT73 and wild-type ewe samples in the cerebellum. None of the 11 metabolites was differentially abundant between OVT73 and wild-type ram samples alone in the cerebellum (Table 4).

### Differential Abundance of Single Metabolites in the OVT73 Liver

Transgene effects were also detected in the liver, where the average relative abundance of 15 metabolites differed significantly between OVT73 and wild-type (Table 3). All of these metabolites were more abundant in OVT73 samples than wild-type, with the exception of succinic acid which was 27.2% lower in the transgenics. Eleven of the 15 nominally differentially abundant compounds in the liver were fatty acids (Table 3). Differences in the relative abundance of 3 metabolites; dodecanoic acid (77%), myristic acid (101%), and nicotinic acid (21%), remained significant after FDR adjustment for multiple testing (Fig. 1).

The 15 metabolites that were nominally differentially abundant in the OVT73 liver were subsequently tested for sex effects. All 15 metabolites remained differentially abundant (*P* < 0.05) in a statistical model where the variable of transgene status was nested within the variable of sex. Indeed, *P*-values for 12 of the 15 metabolites were more significant in the nested model than for the transgene effect alone (Table 4). Examining the effect of transgene status for each sex respectively, all 15 metabolites were differentially abundant between OVT73 and wild-type ram samples in the liver. Comparing the same 15 metabolites between OVT73 and wild-type ewe liver samples, only alanine was differentially abundant (Table 4).

### Metabolite-Metabolite Correlations are Stronger in OVT73 Tissues than Wild-Type

Many metabolites are known to be co-regulated as part of metabolic networks and therefore their abundances will correlate^22^. Based on this knowledge we further predicted that metabolite regulation may be altered in the OVT73 sheep. To explore this hypothesis, Pearson correlations were generated for all possible metabolite-pairs and compared between wild-type and OVT73. Metabolites whose abundances had a strong correlation coefficient (r) were considered likely to be co-regulated.

**Figure 2:**
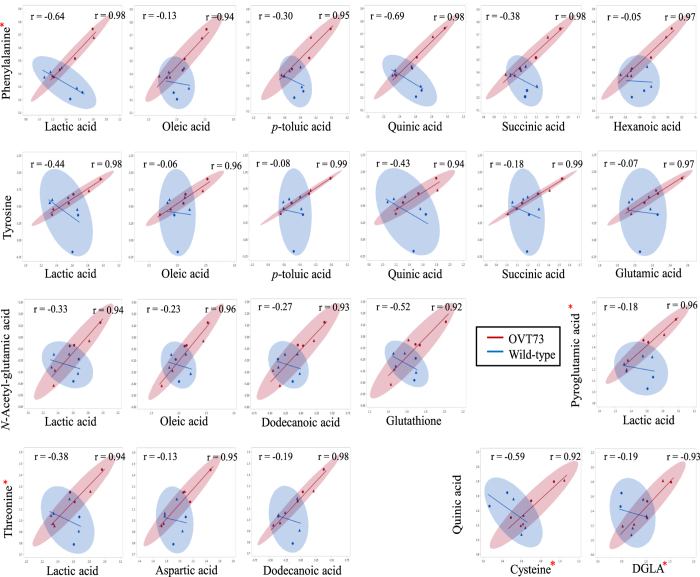
Transgene-Altered Metabolite Correlations in the OVT73 Cerebellum Scatterplots display the correlation of the abundance of the 22 metabolite-pairs that had a transgene-altered correlation in the cerebellum (*P* < 0.05, Fisher r-to-z-transformation). The Pearson correlation coefficient (r) for wild-type (top left) and OVT73 (top right) is shown for each metabolite pair. Metabolite abundances are normalised to 2,3,3,3-d_4_-alanine internal standard, duplicate measurements for each sample averaged and expressed as log10-transformed arbitrary units. Wild-type (n = 6) is shown in blue, transgenic (n = 6) is shown in red. Ellipsoids are the 95% confidence interval for correlations. Triangles are rams, circles are ewes *Metabolites which were also differentially abundant (*P* < 0.05, Standard Least Squares).

**Figure 3:**
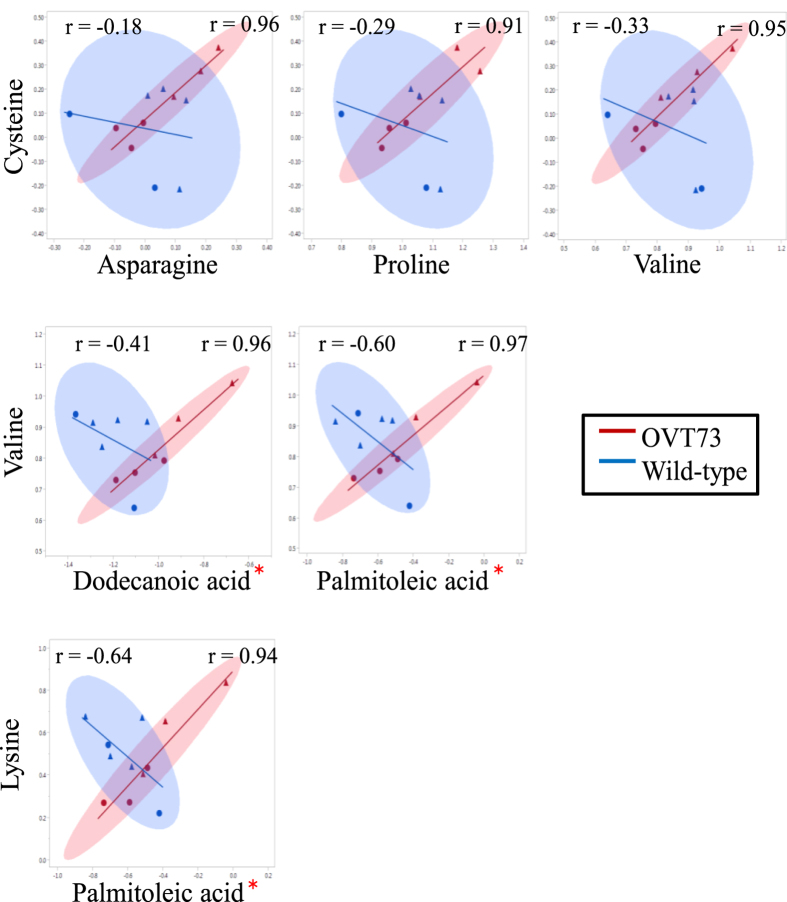
Transgene-Altered Metabolite Correlations in the OVT73 Liver Scatterplots display the correlation of the abundance of the 6 metabolite-pairs that had a transgene-altered correlation in the liver (*P* < 0.05, Fisher r-to-z-transformation). The Pearson correlation coefficient (r) for wild-type (top left) and OVT73 (top right) is shown for each metabolite pair. Metabolite abundances are normalised to 2,3,3,3-d_4_-alanine internal standard, duplicate measurements for each sample averaged and expressed as log10-transformed arbitrary units. Wild-type (n = 6) is shown in blue, transgenic (n = 6) is shown in red. Ellipsoids are the 95% confidence interval for correlations. Triangles are rams, circles are ewes. *Metabolites which were also differentially abundant (*P* < 0.05, Standard Least Squares).

We first identified the number of metabolite-pairs that were strongly correlated; defined as r > 0.9 or <−0.9 (*P* < 0.0001). Strong correlations were observed in all 4 tissues and many of the metabolite-pairs with strong correlations in wild-type were also strongly correlated in OVT73 (Table 5). Surprisingly, this analysis also revealed that the OVT73 samples had more than twice as many strongly correlated metabolite-pairs than wild-type (Table 5). To quantify this phenomenon we determined the statistical significance of differences in the strength of metabolite-pair correlations between wild-type and OVT73 samples. Nominally significant differences (*P* < 0.05) were deemed ‘transgene-altered’ correlations. In total, 22 transgene-altered correlations were detected in the cerebellum (Fig. 2), 6 in the liver (Fig. 3) and none in the hippocampus or motor cortex GC-MS datasets. In all instances the metabolite-pairs with a transgene-altered correlation were strongly correlated in OVT73 samples but weakly correlated in wild-type.

Some metabolites had a transgene-altered correlation with more than one other metabolite. In the liver for example the metabolite cysteine had a transgene-altered correlation with asparagine, proline and valine. These ‘nodes’ of transgene-altered correlation were apparent in both liver and cerebellum datasets (Figs 2 and 3).

## Discussion

We undertook a comparative study of metabolites in our pre-manifest sheep model of HD in order to gain insight into the disease process. The major effects will be discussed first, followed by consideration of the individual metabolites and specific correlations affected.

The OVT73/wild-type differential abundance of single metabolites was determined in post-mortem cerebellum, motor cortex, hippocampus and liver from 5 year old sheep. Eleven metabolites with nominally significant changes in abundance were observed in the cerebellum, 15 in the liver and none in the motor cortex or hippocampus. The differences in cerebellum and liver provide the first indication of a metabolic defect in the OVT73 sheep and further evidence for a dysfunction of metabolism in tissue expressing human mutant huntingtin. All except one of the metabolites (succinic acid in the liver) were more abundant in the transgenic samples than wild-type, suggesting that the alterations are part of a general shift in metabolic activity.

The differential abundance of metabolites in the transgenic sheep also showed remarkable tissue specificity. Nine of the 11 nominally altered metabolites in the cerebellum were amino acids/amino acid derivatives, while 11 of the 15 affected metabolites in the liver were fatty acids. Cerebellar pathology has not been considered a hallmark of the HD mutation, but is seen in juvenile-onset HD^23^ and recent studies have also shown clear Purkinje cell loss in the cerebellum of adult-onset cases^24^. Effects of the mutation in human liver have received little attention to date, although progressive hepatic mitochondrial dysfunction was recently reported in individuals with both pre-manifest and manifest HD^25^,^26^. Our findings highlight the importance of considering HD as a whole body disorder. Moreover, further evidence for a systemic dysfunction opens the possibility that treatment targeted at peripheral tissues may be beneficial in HD.

Metabolite abundance changes in the OVT73 sheep also appeared to be sex-specific. All 11 metabolites that were nominally altered in the mixed-sex cerebellum were, upon single-sex analysis, found to be significantly altered in the transgenic ewes but not the rams. In the liver by contrast, 14 of the 15 nominally altered metabolites in the mixed-sex group, were significant only in the transgenic rams. These observations are striking, although should be interpreted with caution due to the small sample size of the single-sex groups. Sex differences in HD have not been fully characterised to date. Metabolite levels in healthy human populations are influenced by sex however^27^,^28^ and therefore it is reasonable that the metabolic response of males and females to disease may also differ.

We also report differential correlations between pairs of metabolites, suggesting an altered pattern of metabolite regulation in the OVT73 sheep. Pair-wise correlation analysis has previously been used as a method for metabolic fingerprinting and identifying specific points in pathways that are critical for the metabolic regulation in different conditions^22^. As expected, we identified strong correlations (r > 0.9) in both wild-type and transgenic samples, and many were in common between disease groups. Surprisingly, there were many more strong metabolite-pair correlations in the transgenic samples than wild-type for all four tissues. This observation may indicate that metabolism in the OVT73 sheep is under different constraints than in wild-type.

A feature of the correlation data was ‘nodes’ of transgene-altered correlations in the sheep liver and cerebellum; where one metabolite had a transgene-altered correlation with two or more other metabolites. In the liver for example, cysteine had a transgene-altered correlation with three other metabolites (asparagine, isoleucine and valine). The existence of nodes of transgene-altered correlation suggests that metabolite regulation in the OVT73 sheep has been altered in a co-ordinated pattern. Discriminating cause from effect of the abundance and regulation changes for these metabolites requires further investigation and may highlight metabolite pathways directly influenced by mutant huntingtin.

After correction for multiple testing, pyroglutamic acid and phenylalanine were found to be significantly more abundant in the OVT73 cerebellum, and dodecanoic acid, myristic acid and nicotinic acid were significantly more abundant in the OVT73 liver. The greater abundance of phenylalanine and pyroglutamic acid is intriguing as these are features of two metabolic diseases with neurological phenotypes; Phenylketonuria and 5-oxoprolinuria respectively. In Phenylketonuria mutations in the phenylalanine hydroxylase gene (OMIM Entry-# 261600) result in elevated phenylalanine, with intellectual disability and seizures being characteristic of the disease^29^. In 5-oxoprolinuria (OMIM Entry-# 266130) the mutation of genes encoding enzymes involved in glutathione synthesis cause an elevation of pyroglutamic acid and symptoms which include mental retardation, seizures and ataxia. The size of differences in phenylalanine and pyroglutamic acid levels in the transgenic sheep, if seen in humans, would not cause these diseases. It is feasible however that elevated levels of either metabolite could contribute to at least some of the known pathology and symptoms of HD. It will therefore be interesting to monitor these differences as the sheep age. Notably, Phenylketonuria can be effectively managed by restricting phenylalanine in the diet^29^. Alkali substances and antioxidants such as vitamins C and E may also improve prognosis in 5-oxoprolinuria^30^.

A potentially confounding aspect to this study as it relates to predicting CAG-associated changes in specific metabolites in humans is the differences in ruminant metabolism. Like humans, ruminants utilise glucose as their primary energy source, but unlike humans, very little glucose is absorbed from the ruminant diet. Instead, ruminants absorb short carbon chain saturated fatty acids from the diet e.g. acetic acid, propionic acid and butyric acid, and the liver uses these to generate glucose by gluconeogenesis^31^. Free fatty acid uptake by the liver is proportional to plasma concentrations^32^ and the ruminant liver does not tend to synthesise or store fat^33^,^34^. The specific accumulation of medium – long carbon chain fatty acids in the OVT73 sheep liver may therefore reflect their delivery from other tissues and/or impaired clearance from the liver. The differentially abundant metabolites in the liver were both saturated and unsaturated. Unsaturated fatty acids are not typically absorbed from the rumen^35^ so their accumulation in the transgenic sheep liver may reflect mobilisation of stored fat from adipose tissue. Interestingly, the increase in nicotinic acid in the transgenic liver could be a response to the increased fatty acid level, due to its known anti-lipolytic function^36^. Fat mobilisation typically occurs in response to negative energy balance, caused for example by fasting^33^. Energy intake was not measured in this study, but the transgenic and wild-type sheep were managed under identical conditions. This result could therefore indicate a hyper-metabolic phenotype in the OVT73 sheep; consistent with conclusions from other metabolic studies of HD^4^,^10^,^11^. Importantly, the detection of metabolite changes in advance of symptoms raises the possibility that dietary intervention could alter the disease onset and/or progression. The OVT73 sheep are an appropriate testing system for such an intervention due to their well-controlled diet and environment.

This proposed hyper-metabolic state in the transgenic sheep is supported by the amino acid abundance differences detected in the cerebellum. Three of the affected metabolites; phenylalanine (significant after FDR adjustment), valine and threonine (nominally significant), are essential amino acids which can only be obtained through the diet^37^. Assuming equal energy intake in the wild-type and transgenic sheep because they were fed *ad libitum* in the same environment, the higher abundance of essential amino acids in the OVT73 cerebellum may indicate a catabolic state. It is important to note however that we have not observed differences in brain volume in the transgenic animals to indicate overt neurodegeneration^18^. Blood metabolite studies have reported lower levels of specific amino acids in HD patients^4^,^5^,^10^. Metabolite concentrations in blood reflect the complex balance of many tissue processes, e.g. rates of protein catabolism compared to amino acid uptake for protein synthesis. Mochel and colleagues hypothesised that plasma amino acids may be reduced due to their compensatory uptake into tissues to provide substrates for the citric acid cycle, which is thought to be deficient in HD^10^. Our data may also indicate a deficiency in the citric acid cycle, with lower levels of succinic acid; a key intermediate of the citric acid cycle, observed in the OVT73 liver.

Correlating the abundances of pairs of metabolites is considered a sensitive method for determining systemic changes in regulation^22^. Using this approach we identified statistically significant transgene-altered correlations in the sheep liver and cerebellum. Several metabolites with a transgene-altered correlation were also differentially abundant between wild-type and transgenics, i.e. phenylalanine, pyroglutamic acid and cysteine in the cerebellum; oleic acid, dodecanoic acid and palmitoleic acid in the liver. However, the majority of metabolites with a transgene-altered correlation did not show absolute transgene-associated changes in abundance. It is possible that the altered co-regulation may have enabled homeostatic levels of these metabolites to be maintained. Such molecular adaptation could be protective and explain why the OVT73 sheep are outwardly indistinguishable from wild-type.

In summary, the single metabolite differences and altered metabolite co-regulation observed in this study indicate a metabolic defect in the transgenic sheep model of HD. Our findings provide leads for validation and functional investigation of this defect. Further experimentation may provide insight into molecular mechanisms of HD and potentially reveal targets for therapeutic intervention that are relevant to the pre-manifest period of the disease.

## Methods

### Animals Assessed

Tissue samples were obtained from the available 6 wild-type (2 ewes, 4 rams) and 6 OVT73 (3 ewes, 3 rams) sheep aged 5 years. All animals were maintained at the South Australian Research and Development Institute (SARDI) in accordance with the SARDI/PIRSA Animal Ethics Committee (Approval number 19/02, and necropsies performed under 05/12). The animals were kept as part of mixed wild-type/transgenic flocks in large paddocks typical for South Australian farming conditions, and grazed pasture ad libitum, with feed supplementation during dry periods. Rams and ewes were kept in separate flocks. Sample details for the 12 animals, including body weight, brain weight, and sampling delay (time from death to completion of tissue sampling) are provided (Table 1). There were no significant differences between wild-type and transgenic for brain weight, body weight or sampling time. Anticipated sex differences were noted, i.e. on average rams were heavier (91.7 ± 10.5 kg; mean ± SD) than ewes (76.8 ± 5.8 kg) (*P* = 0.02; Standard Least Squares) and the sampling time for rams (58.4 ± 4.9 min) was 12 minutes longer than for ewes (46.2 ± 4.0 min) due to the thicker skull of rams (*P* = 0.001; Standard Least Squares).

Transgenic status was confirmed by PCR amplification of the transgene from tail-tissue genomic DNA using methods described previously^16^. The transgene repeat length was relatively stable at 73 units in DNA isolated from the striatum of all 6 OVT73 sheep. The transgene-derived human huntingtin transcript and protein were expressed as expected in brain and peripheral tissues. Semi-quantitative western analysis reveals negligible differences in transgene expression levels between brain regions (previously quantified by TR-FRET at 31 ng/mg tissue in occipital pole), but markedly lower expression in the liver as expected^17^ (S.J. Reid, Personal Communication). Huntingtin-positive neuropil aggregates have been identified in all brain regions analysed to date (piriform cortex, somatosensory cortex, motor cortex and striatum) from all 6 transgenic animals studied, with a similar number of aggregates (4–6 per mm^2^ on average) detected in each region^38^. Neuropathological analysis has not been undertaken on hippocampus, cerebellum, or liver from the sheep.

### Sample Preparation

Sheep were killed by rapid intravenous injection of pentobarbitone sodium solution (Lethabarb, 1 mL/2 kg body weight) and brain and peripheral tissues were immediately sampled and snap frozen in liquid nitrogen. All experimental protocols were approved by the University of Auckland Animal Ethics Committee and the SARDI/PIRSA Animal Ethics Committee (Approval number 19/02). For experiments described here tissue was available from the liver and three brain regions; motor cortex, hippocampus, and cerebellum, and fresh frozen in liquid nitrogen. Striatum was not included in the experiment because there was insufficient tissue. Approximately 300 mg of each frozen tissue sample was crushed into a fine powder using a mortar and pestle, under liquid nitrogen. Metabolic processes were quenched and small, polar metabolites extracted from the powdered samples using a cold methanol-based method described by Villas-Boas and colleagues^33^. Immediately before metabolite extraction 0.4 μmol of the internal standard 2,3,3,3-d_4_-alanine (Sigma Aldrich; St Louis, USA) was added to each sample to enable variation in the efficiency of metabolite extraction between samples to be accounted for in downstream analysis. The tissue pellet remaining from each sample after metabolite extraction was incubated at 37 °C until dry and then weighed in order to account for variation in the starting amount of each sample. Each metabolite extraction was split into two aliquots which were derivatised separately as technical replicates, using a methyl-chloroformate method previously described^33^.

### Gas Chromatography-Mass Spectrometry

Methyl-chloroformate derivatives were assessed using an Agilent 7890A gas chromatograph coupled to a 5975C inert mass spectrometer. Instrument parameters were based on those previously described^33^. The duplicate sets of samples were analysed sequentially. Following GC-MS, metabolites were identified and quantified using *Metab*; an R package for automated analysis of GC-MS data^34^. *Metab* can identify a set of approximately 165 metabolites that have well described mass spectra. The raw abundance of each named metabolite was determined as the height of its designated major mass fragment. GC-MS and *Metab* analysis were performed by the University of Auckland Centre for Genomics, Proteomics and Metabolomics (Auckland, NZ).

### Statistical Analysis

The raw abundance of each metabolite was normalised to the abundance of the internal standard 2,3,3,3-d_4_-alanine and to the weight of the dried tissue pellet for the same sample. Relative abundance values were log_10_ transformed to approximate a normal distribution.

Differences in the relative abundance of single metabolites between OVT73 and wild-type samples were tested for significance using a standard least squares linear model (JMP^®^ 11.2.0 10). Technical replicates of each sample were included additively in the model and differences in relative abundance at *P* < 0.05 are reported. Multiple testing adjustments (False discovery rate; FDR) were subsequently applied and differences remaining significant at *P* < 0.05 after adjustment are indicated.

Due to the small sample number (wild-type n = 2 ewes, 4 rams; OVT73 n = 3 ewes, 3 rams) we did not test for sex differences in the abundance of metabolites; however metabolites whose abundance differed between wild-type and OVT73 overall at the nominal significance level (*P* < 0.05) were subsequently tested for sex-specificity of the transgene effect. This was achieved by nesting the variable of transgene status within the variable of sex, in the statistical model (Standard Least Squares; JMP^®^ 11.2.0 10).

To investigate the co-regulation of metabolites, Pearson correlations were generated for each possible metabolite pair (JMP^®^ 11.2.0 10). Relative abundance values for technical replicates were averaged prior to analysis. The data was examined for correlations that were strong in one group but weak in the other, to identify differential correlations between OVT73 and wild-type samples. A strong correlation was defined as having a correlation coefficient (r) >0.9 or <−0.9 and that was statistically significant at the level of *P* < 0.05; a weak correlation was defined as r < 0.1 or >−0.1 with *P* > 0.05. These nominal coefficients were conservative to reflect the small sample size. Correlations meeting these criteria underwent a simple bootstrap analysis to identify and remove those caused by outliers. This involved the repeated generation of Pearson correlations, each time removing a single wild-type and single OVT73 sample. For an initial correlation of r > 0.9 or <−0.9 to validate, the threshold for each bootstrap analysis was set at r > 0.7 or <−0.7 respectively with *P* < 0.05. To validate a lack of correlation where the initial correlation was r < 0.1 or >−0.1, a threshold for each bootstrap analysis was set to r < 0.2 or >−0.2. Relaxed thresholds were applied to account for loss of power due to the reduced sample size. For correlations meeting these criteria, the statistical significance of differences in correlation coefficient between OVT73 and wild-type were tested by Fisher R-to-Z transformation (calculator available at http://www.vassarstats.net/rdiff.html). Differences were declared significant at *P* < 0.05.

## Additional Information

**How to cite this article**: Handley, R. R. *et al.* Metabolic disruption identified in the Huntington’s disease transgenic sheep model. *Sci. Rep.* **6**, 20681; doi: 10.1038/srep20681 (2016).

## Figures and Tables

**Table 1 t1:** Sheep Sample Details.

Animal ID	Status	Sex	Age (y, m)	Body Weight (kg)	Brain Weight (g)	Sampling delay (min)
1	Wild-type	Ewe	5y, 4m	76.4	128.9	52
2	Wild-type	Ewe	5y, 4m	86.6	132.5	41
3	Wild-type	Ram	5y, 10m	94.4	126.1	62
4	Wild-type	Ram	5y, 10m	74.2	123.7	50
5	Wild-type	Ram	5y, 10m	104.0	138.6	56
6	Wild-type	Ram	5y, 10m	104.0	143.7	63
7	OVT73	Ewe	5y, 4m	72.0	124.2	47
8	OVT73	Ewe	5y, 4m	75.6	128.2	45
9	OVT73	Ewe	5y, 4m	73.2	118.2	46
10	OVT73	Ram	5y, 10m	85.8	124.6	61
11	OVT73	Ram	5y, 10m	88	123.8	55
12	OVT73	Ram	5y, 4m	91.4	129.2	62

The transgene status and sex of each sheep used in this study is presented. Parameters recorded at death are detailed: age in years (y) and months (m), body weight in kilograms (kg), brain weight in grams (g) and sampling delay as minutes (min) from death until all tissues were dissected and frozen. Refer to Materials and Methods for group statistics.

**Table 2:** Composition of the Sheep GC-MS Metabolite Profiles.

Tissue	Amino acids/derivatives (%)	Fatty acids (%)	Organic acids (%)	Total (%)
Cerebellum	41.2	45.1	13.7	100
Hippocampus	51.4	32.4	16.2	100
Motor cortex	52.1	37.5	10.4	100
Liver	47.9	33.3	18.8	100

The metabolites analysed in this study were classified as amino acid/amino acid derivatives, fatty acids and organic acids respectively. The number of metabolites within each compound class is presented as a percentage (%) of the total number of metabolites identified and quantified in all 12 samples (both genotypes and sexes) by gas-chromatography mass spectrometry, for each tissue respectively.

**Table 3:** Differentially Abundant Metabolites in the OVT73 Sheep Cerebellum and Liver.

Metabolite	OVT73	Wild-type	SED	*P* value	Metabolite class
Cerebellum					
Pyroglutamic acid*	1.42	1.20	0.04	0.001 (0.034)	Amino acid derivative
Phenylalanine*	0.52	0.33	0.04	0.001 (0.034)	Amino acid
Glycine	1.06	0.88	0.04	0.007	Amino acid
Serine	0.51	0.28	0.06	0.008	Amino acid
Cysteine	0.73	0.51	0.05	0.009	Amino acid
2-aminoadipic acid	−0.21	−0.46	0.06	0.014	Amino acid derivative
Dihomo-γ-linolenic acid (C20:3)	1.03	0.72	0.09	0.017	Fatty acid (unsaturated)
Proline	1.00	0.86	0.04	0.020	Amino acid
Valine	0.93	0.80	0.04	0.021	Amino acid
Threonine	1.17	0.99	0.05	0.025	Amino acid
Docosahexaenoic acid (C22:6)	2.11	1.85	0.08	0.035	Fatty acid (unsaturated)
Liver					
Dodecanoic acid (C12:0)*	−0.98	−1.2	0.04	0.001 (0.017)	Fatty acid (saturated)
Myristic acid (C14:0)*	−0.07	−0.35	0.053	0.001 (0.017)	Fatty acid (saturated)
Nicotinic acid*	−0.31	−0.39	0.02	0.001 (0.017)	Amino acid derivative
Pentadecanoic acid (C15:0)	−0.19	−0.40	0.05	0.005	Fatty acid (saturated)
Oleic acid (C18:1)	1.06	0.92	0.04	0.015	Fatty acid (unsaturated)
Alanine	1.57	1.47	0.03	0.017	Amino acid
Succinic acid	0.77	0.90	0.04	0.023	Organic acid
2-aminoadipic acid	−0.66	−0.76	0.03	0.027	Amino acid derivative
Palmitic acid (C16:0)	1.24	1.11	0.04	0.028	Fatty acid (saturated)
Dihomo-γ-linolenic acid (C20:3)	−0.49	−0.66	0.05	0.030	Fatty acid (unsaturated)
Arachidic acid (C20:0)	−0.97	−1.12	0.05	0.033	Fatty acid (saturated)
*trans*-9-Heptadecenoic acid (C17:1)	−0.33	−0.49	0.05	0.038	Fatty acid (unsaturated)
Docosahexaenoic acid (C22:6)	0.39	0.21	0.06	0.039	Fatty acid (unsaturated)
Heptadecanoic acid (C17:0)	0.49	0.32	0.06	0.041	Fatty acid (saturated)
Palmitoleic acid (C16:1)	−0.46	−0.63	0.06	0.042	Fatty acid (unsaturated)

Mean abundance and the standard error of the difference (SED) are presented for metabolites that were differentially abundant in OVT73 (n = 6) compared with wild-type (n = 6) (*P* < 0.05; Standard Least Squares). Data presented are normalised to the abundance of 2,3,3,3-d_4_-alanine internal standard and expressed as log 10-transformed arbitrary units. * Metabolites that were differentially abundant (*P* < 0.05) after correction for multiple testing. False discovery rate adjusted *P* values are reported in brackets for these metabolites.

**Table 4:** Sex-Specificity of Transgene Effects on Metabolite Abundance in the OVT73 Cerebellum and Liver.

Metabolite	Transgene Model	Nested model		
	Overall	Overall	Ewe [Transgene]	Ram [Transgene]
Cerebellum				
Pyroglutamic acid	0.001	<0.0001	<0.0001	0.347
Phenylalanine	0.001	0.002	0.0002	0.136
Glycine	0.007	0.010	0.003	0.330
Serine	0.008	0.005	0.001	0.476
Cysteine	0.009	0.008	0.004	0.110
2-aminoadipic acid	0.014	0.001	0.018	0.283
Dihomo-γ-linolenic acid (C20:3)	0.017	0.003	0.0003	0.821
Proline	0.020	0.073	0.025	0.315
Valine	0.021	0.002	0.0002	0.998
Threonine	0.025	0.006	0.0007	0.908
Docosahexaenoic acid (C22:6)	0.035	0.013	0.001	0.835
Liver				
Dodecanoic acid (C12:0)	0.001	0.0004	0.089	0.0001
Myristic acid (C14:0)	0.001	<0.0001	0.94	<0.0001
Nicotinic acid	0.001	0.004	0.095	0.005
Pentadecanoic acid (C15:0)	0.005	0.0004	0.733	<0.0001
Oleic acid (C18:1)	0.015	0.012	0.975	0.001
Alanine	0.017	<0.0001	0.007	0.0006
Succinic acid	0.023	0.025	0.391	0.007
2-aminoadipic acid	0.027	0.045	0.286	0.017
Palmitic acid (C16:0)	0.028	0.027	0.941	0.003
Dihomo-γ-linolenic acid (C20:3)	0.030	0.008	0.672	0.0009
Arachidic acid (C20:0)	0.033	0.009	0.623	0.001
*trans*-9-Heptadecenoic acid (C17:1)	0.038	0.035	0.922	0.004
Docosahexaenoic acid (C22:6)	0.039	0.018	0.644	0.003
Heptadecanoic acid (C17:0)	0.041	0.011	0.586	0.003
Palmitoleic acid (C16:1)	0.042	0.006	0.723	0.0008

The statistical significance of transgene effects on metabolite abundance is compared between two statistical models. The initial *Transgene Model* tested for effects of the variable of transgene status (OVT73 vs. WT) on metabolite abundance. There were 11 differentially abundant metabolites in the cerebellum and 15 in the liver (*P* < 0.05, Standard Least Squared). This subset of metabolites was analysed for sex-specificity of the transgene effect using a *Nested Model,* with significant effects observed (*P* < 0.05; Standard Least Squares). Analysis for transgene effects within single-sex groupings, as indicated by *Ewe [Transgene]* and *Ram [Transgene]* columns, indicates sex-specificity of the transgene effects on metabolite abundance.

**Table 5:** Strongly Correlated Metabolite-Pairs in Wild-Type and OVT73 Sheep Brain and Liver Tissues.

Tissue	Wild-type (r > 0.9)	OVT73 (r > 0.9)	Shared (r > 0.9)	Total correlations (each status)
Cerebellum	250	101	206	1275
Motor cortex	138	257	127	1128
Hippocampus	39	86	21	666
Liver	60	132	40	1128

The number of ‘strong’ metabolite-pair correlations (r > 0.9/ < −0.9) in wild-type and OVT73 groups is compared for each GC-MS dataset (cerebellum, motor cortex, hippocampus and liver). The number of metabolite pairs with a strong correlation (r > 0.9/ < −0.9) in both wild-type and OVT73 groups) is detailed in the ‘shared’ column. The total number of all pair-wise correlations generated for each dataset is provided in the far right column. Pearson correlations were generated using duplicate averaged and log_10_ transformed relative metabolite abundances.
